# Supplementary material for: Learning curve and surgical outcome of robotic assisted colorectal surgery with ERAS program
Source: Sci Rep. 2022 Nov 29;12:20566. doi: 10.1038/s41598-022-24665-w (PMC9709162; doi:10.1038/s41598-022-24665-w)
Supplement: Supplementary file 1 — Supplementary Information 1. [file 41598_2022_24665_MOESM1_ESM.zip › LSC Raw data-Ñ[▒K/127/O_ñw╝╨░O▒Kñσ.pdf]

正式摘要(電子簽章：000000218639643)

請選擇...reporter

臺中榮民總醫院

Operation Note

[redacted]

- 手術日期：2021/06/05
- 手術醫師：VS 林俊余，CR 楊曙榮，R 陳琦夫
- Anesthesia : GE
- Position : Supine
- Aseptic agents : Soap , Betadine Alcohol , Betadine Aqua

Diagnosis

- Pre-operative diagnosis : A-colon diverticulysis with abscess formation
- Post-operative diagnosis : Ditto, s/p lapascopic RH

Surgical Indication

- RLQ pain with tenderness

Operation

- Laparoscopic right colectomy and anastomosis

Finding

- 1. one diverticulytis at A-colon with abscess and localization
  2. 12mm penrose drain left at pelvic
  3. Hylobarrier +

Procedure

- Under GE anestheia, the patient was put in supine position. GS table consult for A-colon diverticulytis and pus formation. 12mm trocar was made over LUQ and 5mm trocar was made over RLQ. The Ileocolic vessels was dissected, skeletonized and transectioned by clipping and Harmonic scalpel. The retroperitoneal dissection was done through embryonic plane between meso-right colon and Gerota fascia. The Duodenum and pancrease head was identified and protected. Finally, the right side colon was fully mobilized. Extracoporeal resection of ileum and A-colon with side to side anastomosis was done by endo GIA 60/3.8mm X3. 12mm penrose drain inserted to thwe pelvic and the umbilical wound was closed in layers. Throughout the whole course of operation, little blood loss and patient stood well.
- Estimated blood loss: minimum
- 器械數量: 正確
- 紗布數量: 正確

Image

- PAINT
  - abdomen

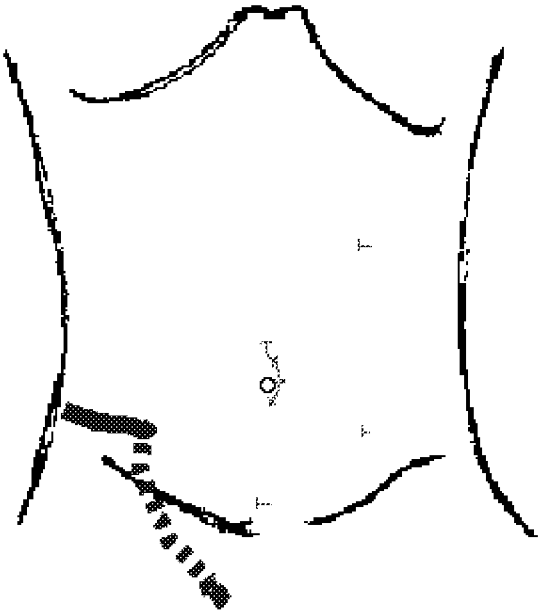

- colon & rectum

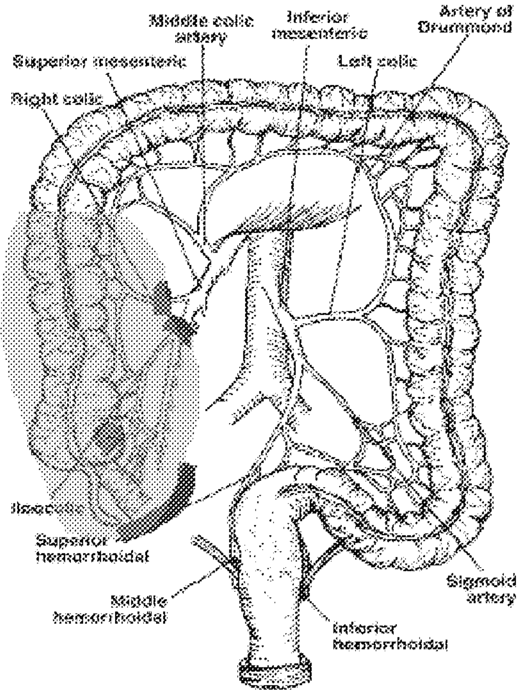

主治 2222H林俊余 / 醫師 2292G楊曙榮

正式摘要(**電子簽章：000000218639643)**

請選擇...reporter

doctorRank:B
